# Efficacy of DC-CIK Immunotherapy Combined with Chemotherapy on Locally Advanced Gastric Cancer

**DOI:** 10.1155/2022/5473292

**Published:** 2022-07-12

**Authors:** Guiyuan Liu, Dehu Chen, Xiaojun Zhao, Xiaolan You, Chuanjiang Huang, Zhiyi Cheng, Xunan Mao, Haihua Zhou

**Affiliations:** Department of General Surgery, Taizhou People's Hospital, Taizhou, China

## Abstract

The aim of the study is to explore the efficacy and safety of dendritic cell-cytokine-induced killer cell (DC-CIK) immunotherapy combined with chemotherapy in the treatment of locally advanced gastric cancer (LAGC). Among 106 patients with LAGC, 53 received the treatment of oxaliplatin-5-fluorouracil chemotherapy (control group), while the remaining 53 received DC-CIK immunotherapy combined with chemotherapy (DC-CIK group). The short-term efficacy and the changes in immune function indexes (cluster of differentiation (CD)3^+^, CD4^+^, CD8^+^, CD4^+^/CD8^+^, and natural killer (NK) cells) were analyzed. The overall response rate (ORR) was 47.2% (25/53) and 41.5% (22/53), and the disease control rate (DCR) was 69.8% (37/53) and 50.9% (27/53), respectively, in the DC-CIK group and the control group. It could be seen that the ORR had no statistically significant difference between the two groups, while the DCR in the DC-CIK group was significantly better than that in the control group. After treatment, the proportions of CD3^+^ T lymphocytes, CD4^+^ T lymphocytes, CD4^+^/CD8^+^ cells, and NK cells obviously rose, while the proportion of CD8^+^ T lymphocytes obviously declined in the DC-CIK group compared with those in the control group. After treatment, the scores in the function module of the QLQ-C30 scale were greatly higher in the DC-CIK group than those in the control group, while the scores of loss of appetite, constipation, dyspnea, fatigue, pain, and sleep disorders in the symptom module were significantly lower in the DC-CIK group than those in the control group. The median survival time was 23.4 months and 18.6 months, respectively, in the DC-CIK group and the control group. The results of the log-rank test showed that the OS in the DC-CIK group was remarkably superior to that in the control group. DC-CIK immunotherapy combined with chemotherapy can improve the immune cell function, ameliorate the quality of life, and prolong the survival time of LAGC patients, with fewer adverse reactions.

## 1. Introduction

Gastric cancer (GC) is one of the common malignancies in clinic. Currently, surgery and postoperative adjuvant chemoradiotherapy are important treatment means for GC, which can help prolong the survival time of patients. A series of adverse reactions, however, can be caused by chemoradiotherapy, damaging the autoimmune function and reducing the immunity, which not only affects the quality of life of patients but also lowers the sensitivity of patients to chemoradiotherapy and increases the risk of recurrence and metastasis of malignancies [1, 2]. Recently, cellular immunotherapy has emerged as a novel antitumor therapy and raised considerable concern. Cytokine-induced killer (CIK) cells and dendritic cells (DCs) are capable of enhancing the immunity of patients, and they can complement each other's advantages through coculture, which is conducive to promoting the proliferation of CIK cells, further enhancing the ability to recognize tumor cells and the cellular immune response, improving the efficacy of tumor treatment, prolonging the survival time, and ameliorating the quality of life [3–5].

Wang et al. demonstrated that chemotherapy combined with CIK/DC-CIK therapy after surgery improved the prognosis in patients with gastric cancer in a meta-analysis [6]. Du et al. also conducted a meta-analysis and concluded that the combination of CIK/DC–CIK immunotherapy and chemotherapy was a feasible choice to prolong survival and improve the quality of life for patients with advanced GC [7].

In the present study, the clinical efficacy and safety of DC-CIK immunotherapy combined with oxaliplatin-5-fluorouracil chemotherapy in the treatment of locally advanced gastric cancer (LAGC) were explored, and the effect of treatment on the immune function of patients was analyzed, hoping to provide a stronger basis for developing the clinical therapeutic regimen for such patients.

## 2. Materials and Methods

### 2.1. General Data

The clinical data of 106 patients with LAGC treated in Taizhou People's Hospital between June 2017 and May 2019 were collected. The exclusion and inclusion criteria were consistent with a previous study [8]. The inclusion criteria are as follows: (1) patients newly diagnosed with GC by pathological examination, (2) those in TNM stage III, (3) those with measurable lesions ≥1 cm diameter shown on CT or MRI, (4) those with an expected survival time >3 months, (5) those with normal hematopoietic, liver, kidney, and heart functions, and (6) those with an ECOG score of 0–2 points. The exclusion criteria are as follows: (1) patients with severe diseases in the heart, liver, or kidney, (2) those with massive pleural or peritoneal effusion, (3) those with coagulation dysfunction, abnormal blood routine results, or other immune system diseases, or (4) those with a history of mental disease. According to different treatment methods, the patients were divided into the oxaliplatin-5-fluorouracil chemotherapy group (control group, *n* = 53) and the DC-CIK immunotherapy + chemotherapy group (DC-CIK group, *n* = 53). The baseline data of patients before treatment had no statistically significant differences between the two groups (*P* > 0.05) (Table 1). This study was approved by the Ethics Committee of Taizhou People's Hospital (17-TZ#021) and was conducted in accordance with the *Declaration of Helsinki*. All the enrolled patients signed the informed consent.

### 2.2. Treatment Methods

We designed the present study based on the protocol in the previous study [8]. Chemotherapy was performed in the control group. Specifically, oxaliplatin (12 mg/(m^2^·d)) was intravenously dripped on the 1^st^ day, and 5-fluorouracil (500 mg/(m^2^·d)) was intravenously dripped from the 1^st^ day to the 5^th^ day. During treatment, the dose of chemotherapy drugs should be appropriately adjusted according to the patients' tolerance. The treatment lasted for 2 consecutive cycles (21 d as 1 cycle).

In the DC-CIK group, DC-CIK immunotherapy was performed based on the treatment in the control group. After admission, the blood was immediately drawn for the culture of DC-CIK immune cells at 2 d before chemotherapy. The chemotherapy was started from the 3^rd^ day, and the cells cultured maturely were intravenously reinfused. The treatment lasted for 2 consecutive cycles (4 weeks as 1 cycle). During treatment, the patients' adverse reactions were monitored and recorded, and adequate nutritional support was given. The preparation and reinfusion plans of DC-CIK cells are as follows: infectious disease inspections (bacteria, virus, mycoplasma, chlamydia, *etc*.) were strictly performed before blood collection to ensure no bloodstream infection. DC-CIK cells were prepared based on the previous report [9]. The cells were reinfused in the morning and afternoon at 1 week after chemotherapy for the first two times and reinfused in the morning and afternoon at 2 weeks after chemotherapy for the last two times.

### 2.3. Observation Indexes

The short-term clinical efficacy was assessed based on the response evaluation criteria in solid tumors (RECIST), including complete response (CR), partial response (PR), stable disease (SD), and progressive disease (PD).The overall response rate (ORR) = (CR + PR)/total cases × 100%, and the disease control rate (DCR) = (CR + PR + SD)/total cases × 100%.

At 1 week before DC-CIK immunotherapy and 1 month after DC-CIK immunotherapy, the peripheral blood was drawn from each patient, and the content of immune cells, including cluster of differentiation CD3^+^, CD4^+^, CD8^+^, CD4^+^/CD8^+^, and natural killer (NK) cells, was detected by flow cytometry. The adverse reactions during treatment were recorded and assessed in accordance with the National Cancer Institute Common Terminology Criteria for Adverse Events (NCI-CTCAE) v3.0.

The patient's overall function and overall quality of life were assessed using the European Organization for Research and Treatment of Cancer (EORTC) Quality of Life Questionnaire-Core 30 (QLQ-C30) [10]. After treatment, the patients were asked to fill out the questionnaire, based on which their overall function, overall quality of life, and clinical symptoms were scored.

The patients were followed up once every 3 months within 2 years after treatment and once every 6 months after 2 years. The survival time of patients was recorded, and those lost to follow-up were regarded as censored from the date of loss.

### 2.4. Statistical Analysis

Statistical Product and Service Solutions (SPSS) 22.0 (IBM, Armonk, NY, USA) was used for statistical analysis. Measurement data were expressed as the mean ± standard deviation (*χ* ± *s*), and compared by the *t*-test between two groups. The clinical data were compared by the *χ*^2^ test or Fisher's exact probability test. The Kaplan–Meier survival curve was plotted for survival analysis, and the log-rank test was performed. *PP* < 0.05 suggested the statistically significant difference.

## 3. Results

### 3.1. Comparison of Short-Term Efficacy

The efficacy of all patients was assessed after treatment. In the DC-CIK group, there were 2 cases (3.8%) of CR, 23 cases (43.4%) of PR, 12 cases (22.6%) of SD, and 16 cases (30.2%) of PD, and the ORR and the DCR were 47.2% (25/53) and 69.8% (37/53), respectively. In the control group, there were 0 cases of CR, 22 cases (41.5%) of PR, 5 cases (9.4%) of SD, and 26 cases (49.1%) of PD, and the ORR and the DCR were 41.5% (22/53) and 50.9% (27/53), respectively. It could be seen that the ORR had no statistically significant difference between the two groups (*P*=0.696), while the DCR in the DC-CIK group was significantly better than that in the control group, with a statistically significant difference (*P*=0.033) (Table 2).

### 3.2. Comparison of Immunological Indexes between the Two Groups before and after Treatment

There were no statistically significant differences in the proportions of CD3^+^ T lymphocytes, CD4^+^ T lymphocytes, CD8^+^ T lymphocytes, CD4^+^/CD8^+^ cells, and NK cells between both groups (*P* > 0.05). After treatment, the proportions of CD3^+^ T lymphocytes, CD4^+^ T lymphocytes, CD4^+^/CD8^+^ cells, and NK cells obviously rose (*P* < 0.05), while the proportion of CD8^+^ T lymphocytes obviously declined in the DC-CIK group compared with those in the control group (*P*=0.023) (Table 3).

### 3.3. Comparison of Adverse Reactions between the Two Groups

During treatment, no serious adverse reactions occurred in both groups. In the control group, there were 32 cases (60.4%) of fever, 10 cases (18.9%) of hyperpyrexia accompanied by shivering, 3 cases (5.7%) of rash, and 9 cases (17.0%) of myelosuppression. In the DC-CIK group, there were 30 cases (56.6%) of fever, 9 cases (17.0%) of hyperpyrexia accompanied by shivering, 4 cases (7.5%) of rash, and 3 cases (5.7%) of myelosuppression. It could be seen that the incidence rate of fever, hyperpyrexia accompanied by shivering, and rash had no statistically significant difference between the two groups (*P* > 0.05), but the incidence rate of myelosuppression was distinctly lower in the DC-CIK group than that in the control group (*P*=0.022).

### 3.4. Comparison of the Quality of Life Score between the Two Groups after Treatment

The quality of life of patients after treatment was recorded during follow-up. According to the QLQ-C30, the scores of physical function, role function, emotional function, social function, and cognitive function in the function module in the DC-CIK group were significantly higher than those in the control group after treatment (*P* < 0.05). The scores of loss of appetite, constipation, dyspnea, fatigue, pain, and sleep disorders in the symptom module were significantly lower in the DC-CIK group than those in the control group (*P* < 0.05). No statistically significant difference was found in the nausea and vomiting scores between the two groups (*P* > 0.05). The aforementioned results indicated that the symptoms were improved more significantly in the DC-CIK group than those in the control group (Table 4).

### 3.5. Follow-Up Results of Patients' Survival Status

**Figure 1:**
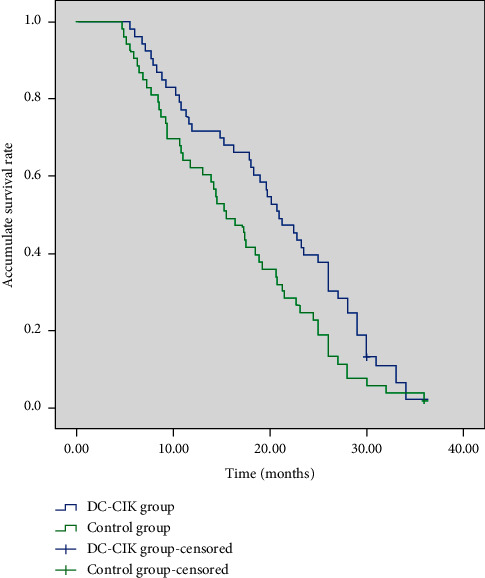
Kaplan–Meier survival curves of locally advanced gastric cancer patients. The overall survival rate of patients in the DC-CIK group was significantly higher than that of the control group (*P*=0.032).

All of the 106 patients were followed up for 3–36 months until May 2021. The median survival time was 23.4 months and 18.6 months, the 1-year overall survival (OS) rate was 71.7% (38/53) and 62.3% (33/53), and the 2-year OS rate was 39.6% (21/53) and 24.5% (20/53), respectively, in the DC-CIK group and the control group. The patient's survival curve was plotted using the Kaplan–Meier method (Figure 1). The results of the log-rank test showed that the OS in the DC-CIK group was remarkably superior to that in the control group (*P*=0.032).

## 4. Discussion

GC is the most common malignancy in the world, whose incidence rate ranks 4^th^ and fatality rate ranks 3^rd^ following lung cancer and liver cancer. Early GC is primarily treated with surgery, but most patients have already been in the advanced stage when diagnosed, losing the opportunity for surgery. Chemotherapy is the major treatment means for advanced GC, but it has disadvantages such as severe toxic and side effects, poor tolerance, and a high rate of treatment discontinuation, affecting clinical efficacy. Moreover, chemotherapy will weaken the body's immunity and reduce the body's killing ability against tumor cells while inhibiting tumor cell proliferation. Besides, conventional chemotherapy can only kill a certain number of tumor cells, but the body's killing ability against small lesions depends on the activation of the autoimmune system [11, 12]. Therefore, it is of great significance to search for a treatment method able to reduce the tumor recurrence rate and raise the survival rate of patients. In the past 2 decades, with the rapid development of tumor immunology and molecular biology, more effective treatment means for cancer have emerged, which display good prospects in improving the prognosis of patients. CIK cells, a cell population with dual antitumor activities of T lymphocytes and NK cells, are characterized by great amplification *in vitro*, high tumor-killing activity, a broad tumor-killing spectrum, and low toxicity to normal tissues [6, 9]. DCs are the only antigen-presenting cells able to significantly stimulate the proliferation of naive T cells, which can trigger the body's adaptive T-cell immune response. Coculture of DC and CIK cells *in vitro* and reinfusion to patients can be used for tumor immunotherapy, and the interaction between DC and CIK cells can induce and ensure an efficient and harmonious immune response [13]. With the rapid development of immunotherapy, cellular immunotherapy has been gradually applied in the treatment of tumors including GC, bladder cancer, and breast cancer [14]. DCs, as important antigen-presenting cells in the body, can release a large number of cytokines such as INF and IL-12, thereby regulating tumor immune response through several ways [15]. Numerous studies have indicated that the immune system of cancer patients is weak, including increased CD3^+^CD8^+^ T cells and decreased CD3^+^CD4^+^ T cells, CD4^+^/CD8^+^ ratio, and NK cells [16]. It is greatly important to activate the immune response against tumor cells for improving the clinical treatment effect. CIK cells are a cell population with antitumor activity, in which CD16^+^CD56^+^ cells can massively release antitumor cytokines and exert great toxicity to tumor cells. Exogenous CIK cells can enhance the body's killing ability against tumor cells, and they also have a good synergistic effect with chemotherapy drugs [17, 18]. In the present study, DC-CIK therapy combined with chemotherapy was given to patients with advanced GC, and its effects on the immune function, survival rate, and quality of life of patients were explored.

In this study, it was found that CD3^+^ and CD4^+^ lymphocytes increased significantly, and the CD4^+^/CD8^+^ ratio rose after treatment, suggesting that the patients' cellular immune function was improved, which was consistent with the previous reports [19, 20]. Meanwhile, the results revealed that DC-CIK therapy combined with chemotherapy could obviously prolong the OS, with mild toxic and side effects, which can be well tolerated. After treatment, the scores of overall function, indexes of the specific symptom module, and the overall quality of life in the QLQ-C30 were significantly higher in the DC-CIK group than those in the control group, but the scores of fatigue, pain, dyspnea, sleep disorders, and loss of appetite were significantly lower in the DC-CIK group than those in the control group (*P* < 0.05). The above results indicated that the symptoms were improved more significantly in the DC-CIK group than those in the control group.

This study was a single-center retrospective study with a small sample size and a short follow-up period. In the future, more rigorous and scientific large-sample prospective multicenter randomized controlled studies should be designed to validate whether the DC-CIK combination therapy can reduce the chemotherapy cycles and whether the risk of postoperative recurrence of GC can be further reduced by increasing the number of DC-CIK cells reinfused and the reinfusion cycles, thereby providing references for selecting the therapeutic regimen for patients with LAGC.

## 5. Conclusion

DC-CIK immunotherapy combined with chemotherapy can improve the immune cell function, ameliorate the quality of life, and prolong the survival time of LAGC patients, with fewer adverse reactions.

## Figures and Tables

**Table 1:** Demographics and general clinical data of all studied patients.

Parameters	DC-CIK group *n* = 53	Control group *n* = 53	*P* value
Gender (male/female)	33/20	27/26	0.327

Age (years)	61.55 ± 9.08	63.10 ± 9.19	0.384

Tumor location			0.782
Cardia of the stomach	6 (11.3%)	5 (9.4%)	
Fundus of the stomach	14 (26.4%)	16 (30.2%)	
Body of the stomach	15 (28.3%)	12 (22.6%)	
Antrum of the stomach	18 (15.1%)	20 (37.7%)	

Pathological type			0.686
Well-differentiated adenocarcinoma	9 (17.0%)	11 (20.8%)	
Moderately differentiated adenocarcinoma	22 (41.5%)	19 (35.8%)	
Poorly differentiated adenocarcinoma	17 (32.1%)	19 (35.8%)	
Mucinous adenocarcinoma	5 (9.4%)	4 (7.5%)	

TNM staging			0.693
IIIA	14 (26.4%)	16 (30.2%)	
IIIB	23 (43.4%)	24 (45.3%)	
IIIC	16 (30.2%)	13 (24.5%)	

ECOG (points)			0.508
0	18 (34.0%)	15 (28.3%)	
1	25 (47.2%)	23 (43.4%)	
2	10 (18.9%)	15 (28.3%)	

*Notes*. DC-CIK: dendritic cell-cytokine-induced killer cells; TNM: tumor, lymph node, metastasis; ECOG: eastern cooperative oncology group.

**Table 2:** Comparison of tumor response of patients in the two studied groups.

Parameters	DC-CIK group *n* = 53	Control group *n* = 53	*P* value
Complete response (CR)	2 (3.8%)	0 (0%)	
Partial response (PR)	23 (43.4%)	22 (41.5%)	
Stable disease (SD)	12 (22.6%)	5 (9.4%)	
Progressive disease (PD)	16 (30.2%)	26 (49.1%)	
ORR (CR + PR)	25 (47.2%)	22 (41.5%)	0.696
DCR (CR + PR + SD)	37 (69.8%)	27 (50.9%)	0.033

*Notes*. DC-CIK: dendritic cell-cytokine-induced killer cells; ORR: overall response rate; DCR: disease control rate.

**Table 3:** Comparison of immunological indicators of patients in the two studied groups.

	DC-CIK group *n* = 53	Control group *n* = 53	*P* value
CD3^+^ T cell (%)			
Pretreatment	55.17 ± 3.73	56.39 ± 4.40	0.127
Post-treatment	60.61 ± 3.63	53.62 ± 3.84	0.001

CD4^+^ T cell (%)			
Pretreatment	33.13 ± 4.09	32.75 ± 4.03	0.631
Post-treatment	36.51 ± 4.47	29.88 ± 4.08	0.001

CD8^+^ T cell (%)			
Pretreatment	27.17 ± 5.14	28.21 ± 5.16	0.301
Post-treatment	26.34 ± 5.19	28.65 ± 5.14	0.023

CD4^+^/CD8^+^ ratio			
Pretreatment	1.43 ± 0.12	1.44 ± 0.13	0.682
Post-treatment	1.56 ± 0.14	1.17 ± 0.13	0.001

NK cell (%)			
Pretreatment	16.52 ± 3.98	17.39 ± 4.09	0.270
Post-treatment	20.19 ± 4.22	15.59 ± 4.49	0.001

Notes: DC-CIK: dendritic cell-cytokine-induced killer.

**Table 4:** Comparison of the postoperative EORTC-QLQ-C30 scale scores of the studied patients in two different groups.

Complications	DC-CIK group *n* = 53	Control group *n* = 53	*P* value
QLQ-C30			
Functioning scales			
Physical	45.62 ± 5.41	42.28 ± 5.18	0.002
Role	47.21 ± 6.59	44.10 ± 6.08	0.013
Emotional	62.73 ± 7.74)	58.96 ± 6.49	0.008
Social	50.71 ± 5.65	48.14 ± 5.90	0.024
Cognitive	65.36 ± 7.05)	62.67 ± 6.55	0.044

Symptom scales			
Appetite loss	40.73 ± 6.15	43.52 ± 7.04	0.032
Constipation	40.07 ± 5.89	43.64 ± 6.35	0.003
Dyspnea	41.34 ± 5.98	44.90 ± 5.09	0.001
Fatigue	40.35 ± 5.26	43.24 ± 6.02	0.010
Nausea and vomiting	21.88 ± 4.48	22.53 ± 4.93	0.479
Pain	23.39 ± 3.75	25.23 ± 3.73	0.013
Sleep disturbance	40.68 ± 6.95	43.68 ± 7.53	0.035

*Note.* EORTC: European Organization for Research and Treatment of Cancer; DC-CIK: dendritic cell-cytokine-induced killer cells.

## Data Availability

The datasets used and analyzed during the current study are available from the corresponding author on reasonable request.
